# The prevalence and incidence of frailty in Pre-diabetic and diabetic community-dwelling older population: results from Beijing longitudinal study of aging II (BLSA-II)

**DOI:** 10.1186/s12877-017-0439-y

**Published:** 2017-02-08

**Authors:** Jagadish Kumar Chhetri, Zheng Zheng, Xitong Xu, Cuihong Ma, Piu Chan

**Affiliations:** 1 Department of Neurobiology, Beijing Institute of Geriatrics, Xuanwu Hospital of Capital Medical University, No. 45 Changchun St., Beijing, Xicheng District 100053 China; 2 Department of Geriatrics, Beijing Institute of Geriatrics, Xuanwu Hospital of Capital Medical University, No. 45 Changchun St., Beijing, Xicheng District 100053 China; 3 Department of Neurology, Beijing Institute of Geriatrics, Xuanwu Hospital of Capital Medical University, No. 45 Changchun St., Beijing, Xicheng District 100053 China; 4 Parkinson’s Disease Center of Beijing Institute for Brain Disorders, Key Laboratory on Neurodegenerative Disease of Ministry of Education, Beijing Key Laboratory for Parkinson’s Disease, Beijing, 100053 China

**Keywords:** Frailty, Elderly diabetes, Pre-diabetes, Elevated- blood glucose

## Abstract

**Background:**

Various factors including cardio-metabolic disorders are found to be correlated with frailty. With the increase in age, older adults are likely to have elevated blood glucose level. In this study we intend to investigate the prevalence and incidence of frailty in the pre-diabetic and diabetic community dwelling elderly population and the associated risk factors.

**Methods:**

At baseline total of 10,039 subjects with a mean age of 70.51 (±7.82) were included. A total of 6,293 older adults were followed up at 12 months. A Frailty index (FI) with 32 items was developed using Rockwood’s cumulative deficits method. Frailty index ≥0.25 was used as cut-off criteria for the diagnosis of frailty. Diagnosis of pre-diabetes and diabetes was set according to the World Health Organization (WHO) criteria for fasting plasma glucose (FPG) level. Chi-square tests were performed to compare percentages by 3 major groups (non-diabetes, pre-diabetes, diabetes), ANOVA and student’s t-tests was used to compare means of group for continuous variables. Multiple logistic regression models were performed to estimate the risk factors for frailty in non-diabetic, pre-diabetic and diabetic elderly populations using baseline and longitudinal data.

**Results:**

Diabetic population had a much higher prevalence (19.32%) and incidence (12.32%) of frailty, compared to that of non-diabetic older adults (prevalence of 11.92% and incidence of 7.04%). And pre-diabetics had somewhat similar prevalence of 11.43% and slightly higher incidence of 8.73% for frailty than non-diabetic older adults. Diabetics were at 1.36 (95% CI = 1.18,1.56) and 1.56 (95%CI = 1.32,1.85) fold increase in risk of frailty compared to non-diabetic population for prevalence and incidence, respectively. Being female, urban living, high waist circumference, less house work and need regular anti-diabetic medications were independent risk factors only in pre-diabetic and diabetic older adults.

**Conclusion:**

This study confirms that diabetes is an independent serious chronic condition to increase the risk of frailty in community dwelling older adults in northern China. To effectively delay or avoid frailty, older adults should be advised for taking proper control of blood glucose level and avoiding the associated risk factors and implementing the protective factors in primary-care setting.

**Electronic supplementary material:**

The online version of this article (doi:10.1186/s12877-017-0439-y) contains supplementary material, which is available to authorized users.

## Background

Ageing is associated with multisystem functional decline, which could lead to future frailty [1, 2]. Various factors including cardio-metabolic disorders are found to be correlated with frailty, among which diabetes in the elderly population cannot be excluded. With the increase in age, older adults are likely to have alteration in glucose metabolism which may be due to decrease in insulin sensitivity and resistance, islet cell dysfunction or reduced beta-cell sensitivity leading to elevated glucose level [3]. Diabetes now a very common chronic disease in the elderly population, itself affects all major organs, therefore affecting multisystem function, which in turn could aid in facilitating acceleration of frailty [4, 5]. Impaired fasting glucose (IFG) or pre-diabetes is a transitional state of diabetes which could also be an associated risk factor for frailty. Previous studies have shown that older diabetic adults are at higher risk of being frail [6–8], however, these study did not examine the risks for frailty in other elevated blood glucose older population. Frailty is a geriatric condition which is known to be reversed or at least delayed, if suitable interventions are implemented on time [9–11]. Moreover, for such interventions to be implemented, the at-risk population and the risk factors should be identified. In this study, we intend to investigate the prevalence and incidence of frailty in community dwelling subjects more than 55 years old with elevated blood glucose (pre-diabetes, diabetes), and if these population shared the common risk factors as normal blood glucose frail population.

## Methods

### Study population

This study is a secondary analyses based on Beijing Longitudinal study of ageing II (BLSA II) [12]. The baseline study was started from July till November 2009 and a follow-up study was performed after a year from August 2010 to January 2011. A multi-stage cluster random sampling method was performed to select aged 55 and older representative community cohort of Beijing residents. Three urban districts and one rural county were selected for this study.

### Data collection

A well designed structured questionnaire was prepared based upon the updated international and domestic guidelines which included socio-economic demographic questionnaires, comprehensive geriatric assessments (CGA) including self-reported history of chronic diseases and performance based functional assessments, history of medications (daily medications within 2 weeks of interview) and existing pre-clinical conditions. A face to face interview was performed by trained clinicians based upon the questionnaire and vital signs, fasting blood glucose, lipids levels, uric acid and hematological test samples were also collected during the same time. The participants were followed and evaluated after 12 months by the same questionnaire. Data inconsistency and missing values were queried and resolved for both visits.

### Measures

We used Rockwood’s accumulation of deficits method [13, 14] to calculate frailty in the BLSA-II study. The frailty index (FI) consisting of 32 items was developed, based upon the methodology introduced by Searle and colleagues [15]. FI ≥ 0.25 was used as the cut-off criteria for frailty [14]. FI items include: 1) 11 chronic conditions, for which the participants were asked “Have you ever been diagnosed for the following conditions by a doctor?” Chronic conditions included hypertension, cardiovascular disease, chronic obstructive pulmonary disease (COPD), stroke, dementia, arthritis, tumor, cataract, deafness, heart failure and renal failure; 2) Four functional assessment scales: a) Mini-Nutrition assessment (MNA), where cutoff was set < 24 as poor nutrition status and ≥ 24 as good nutritional status, b) Tinetti’s performance-oriented assessment of mobility (POMA), where cutoff was set < 24 as poor mobility status and 24 as good mobility status, c) Geriatric Depression Scale-15 items (GDS-15), where cutoff was set < 8 as depressed status and ≥ 8 as normal status, d) Mini-Mental State Examination (MMSE), where cutoff was set < 24 as cognitively impaired state and 24 or more as normal cognitive function; 3) Six disease screening questionnaires (joint pain and inflammation, gout, risk of fall, osteoporosis, arterial sclerosis and Parkinson's disease); 4) Eight age-related symptoms questionnaires (less activity, fatigue, weight loss, urinary inconsistence, fecal inconsistence, memory loss, vision and hearing loss) were included and all participants were screened for these geriatric symptoms. 5) Body mass index (BMI) was calculated by weight/height*height, where BMI <19 was considered to be underweight and 2 blood test results were included for: a) Dyslipidemia: Triglyceride (TG) ≥200 mg/dl or Total Cholesterol (TCH) ≥ 240 mg/dl or low-density lipoprotein cholesterol (LDL) ≥ 160 mg/dl or high-density lipoprotein cholesterol (HDL) < 40 mg/dl). b) Serum uric acid (UA): UA > 420 IU for male, UA > 360 IU for female. Examination of blood sample was done using an automatic biochemical analysis device (Sysmex Chemix-180, Sysmex Infosystems, Kobe,Japan). All included items had less than 5% missing values, and continuous measures were dichotomized into frailty markers (1 = presence,0 = absence) based on the referenced cutoffs for Chinese population [12].

Diagnosis of diabetes and pre-diabetes was set according to the World Health Organization (WHO) criteria of fasting plasma glucose (FPG) and self-history of the patient. Diabetes was defined by WHO criteria of fasting plasma glucose (FPG) greater than or equal to 7.0 mmol/l (126 mg/dl). Impaired fasting glucose (IFG) level of 6.1 to 6.9 mmol/l (110 mg/dl to 125 mg/dl) were taken as “pre-diabetic/pre-diabetes” subjects. Self-history of diagnosed diabetes by doctors at the tertiary hospitals and taking anti-diabetic medications were also considered to be the “diabetic/diabetes” population All other population apart from diabetes and pre-diabetes were taken as “non-diabetic” population. The FPG test was determined using “OneTouch Ultra, Life-Scan, Inc., Milpitas, CA” device during the interview session along with two other blood analyses.

### Statistical analyses

Chi-square tests were performed to compare percentages by three major groups (non-diabetes, pre-diabetes and diabetes), ANOVA and student’s t-tests were used to compare means of the groups for continuous variables. The prevalence and incidence of frailty with 95% confidence interval (CI) were calculated using baseline and follow-up data, and rates of frailty in different demographic and clinical subgroups were estimated. Subgroup analysis was based on demographic conditions (urban or rural residence, gender, age group (55–64years, 65–74years,75–84years, ≥85 years) and clinical conditions including number of co-existing comorbidity (include 11 chronic conditions in the FI) divided into 3 sub-categories (no comorbidity, 1–2, ≥3), polypharmacy (0–3 types/day, ≥4 types/day), anti-diabetic medication (never taken, occasionally, regularly), BMI status (<19, 19–28, ≥28), hours of outdoor activity per day (none,<0.5 hr, 0.5-1 hr,2-3 hrs,≥4 hrs), doing or helping with house work (never, occasionally, regularly). To estimate the association between blood glucose levels with prevalence and incidence of frailty, we calculated unadjusted and adjusted odds ratios (ORs) at baseline and risk ratios (RRs) at one-year follow-up of pre-diabetes and diabetes using single and multiple logistic models adjusted for age, sex, residency and comorbidity.

We then performed stratified analysis to compare associated factors of prevalence and incidence of frailty between elevated blood glucose (pre-diabetes and diabetes) subgroup and normal blood glucose (non-diabetes) subgroup. Backward elimination variable selection method was used and variables initially entered in both models were those included in subgroup analysis and other possible factors like current marital status, low education (less than middle school education was considered low education level), history of smoking and drinking, irregular lifestyle, and high waist circumference (cut-off for Chinese adults male ≥ 85 cm, female ≥ 80 cm) [16]. Estimating the relative risk in cohort studies was based on Deddens 2004 and Zou 2004 [17, 18]. All tests were 2-tailed and P < .05 was considered statistically significant. Multiple comparisons were adjusted using Bonferroni's criteria. All analyses were performed using SAS version 9.3 (SAS Institute, Inc., Cary, NC,U.S.A).

## Results

### Baseline characteristics

Participant’s characteristics at baseline and 12-months follow-up visits are shown in Additional file 1: Table S1 in the Appendix. The mean age of participants at baseline was 70.51 years (SD 7.82) and the majority were women (61.3%), living in urban area (78.4%) and had middle school or higher education (61.45%). Of those, 25.73% participants had no chronic disease, and 20.77% had 3 or more chronic diseases and 22.34% were taking more than four prescription drugs. Considering living habits, only 4.78% had no regular outdoor exercise and 12.35% subjects never performed any household works.

### Prevalence of frailty

At baseline, population without diabetes was 6410, 875 were pre-diabetics and 2634 were diabetics, of which a total of 1373 were frail (Table 1). The highest prevalence of frailty was found in the population with diabetes (19.32%), whereas prevalence of frailty in pre-diabetics (11.43%) and non-diabetes (11.92%) population was quiet similar. The prevalence of frailty was found to be higher in female compared to male in all 3 groups ie non-diabetes, pre-diabetes and diabetes (13.16%, 13.71%, 21.05% vs 9.97%, 8.12%, 16.39%) respectively, urban population were distinctively frail compared to rural (14.51%, 14.70%, 22.25% vs 2.93%, 3.52%, 5.62%) in the 3 groups. The prevalence of frailty was found to increase with age and was highest in oldest of old age group ≥85 (28.83%, 47.62%, 42.31%). Prevalence of frailty increased with the number of co-morbidity in all 3 groups; highest in subjects with 3 or more co-morbidity (43.56%, 45.90%, 49.34%). Similarly, prevalence increased with polypharmacy, subjects with 4 or more medications per day had higher prevalence of frailty (33.24%, 36.67%, 36.19%) in the 3 groups, respectively.

**Table 1:** Prevalence and incidence of frailty by blood glucose status

	Prevalence of frailty				Incidence of Frailty			
	Non-diabetes (*n* = 6410) N (%)	Pre-diabetes (*n* = 875) N (%)	Diabetes (*n* = 2634) N (%)	P value	Non-diabetes (*n* = 4019) N (%)	Pre-diabetes (*n* = 573) N (%)	Diabetes (*n* = 1575) N (%)	*P* value
Overall	764 (11.92)	100 (11.43)	509 (19.32)	<.0001	283 (7.04)	50 (8.73)	194 (12.32)	<.0001
Residence								
Urban	722 (14.51)	91 (14.70)	483 (22.2)	<.0001	247 (7.76)	45 (10.71)	184 (14.10)	<.0001
Rural	42 (2.93)	9 (3.52)	26 (5.62)	0.0254	36 (4.31)	5 (3.27)	10 (3.70)	0.7925
Gender								
Male	249 (9.97)	29 (8.12)	160 (16.39)	<.0001	95 (5.82)	21 (8.57)	61 (9.65)	0.0038
Female	515 (13.16)	71 (13.71)	349 (21.05)	<.0001	188 (7.87)	29 (8.84)	133 (14.10)	<.0001
Age group								
55–64	70 (3.84)	8 (3.00)	51 (8.12)	<.0001	33 (2.76)	8 (4.44)	19 (4.62)	0.1370
65–74	285 (10.51)	41 (11.17)	2013 (17.67)	<.0001	114 (6.36)	23 (9.24)	81 (11.22)	0.0002
75–84	362 (21.17)	41 (18.64)	234 (28.85)	<.0001	116 (12.17)	18 (13.14)	86 (20.33)	0.0003
≥85	47 (28.83)	10 (47.62)	22 (42.31)	0.0712	20 (25.32)	1 (14.29)	8 (42.11)	0.2433
Coexisting co-morbidity								
0	16 (0.78)	1 (0.39)	11 (1.99)	0.0237	42 (3.09)	7 (4.09)	29 (7.55)	0.0005
1–2	356 (10.27)	43 (8.65)	200 (13.55)	0.0007	174 (7.69)	31 (8.88)	95 (9.88)	0.1164
≥3	392 (43.56)	56 (45.90)	298 (49.34)	0.0877	67 (16.92)	12 (22.64)	70 (30.57)	0.0004
Polypharmacy/day								
0–3	409 (7.70)	45 (6.25)	149 (9.19)	0.0353	206 (5.97)	41 (8.47)	116 (10.84)	<.0001
≥4	354 (33.24)	55 (36.67)	359 (36.19)	0.3299	75 (13.69)	9 (10.59)	74 (15.04)	0.5210
Anti-diabetic medication								
Never	/	/	68 (10.86)		/	/	36 (8.82)	0.1794
Occasionally	/	/	11 (22.00)		/	/	5 (15.63)	
Regularly	/	/	430 (21.96)		/	/	153 (13.48)	
BMI status								
<19	58 (22.75)	4 (23.53)	19 (30.65)	0.4275	12 (9.30)	0	1 (2.94)	0.3112
19–28	547 (10.61)	64 (9.61)	373 (18.18)	<.0001	224 (6.85)	34 (7.71)	148 (11.94)	<.0001
≥28	154 (15.88)	32 (17.11)	114 (22.53)	0.0065	47 (7.83)	16 (13.33)	43 (14.68)	0.0040
Hours of outdoor activity/day								
0	93 (32.18)	13 (28.26)	57 (43.18)	0.0545	7 (5.74)	4 (15.38)	4/49 (8.16)	0.2389
<0.5 h	171 (15.21)	26 (16.05)	127 (26.19)	<.0001	51 (7.10)	16 (14.16)	44 (15.88)	<.0001
0.5–1 h	353 (12.32)	46 (13.03)	227 (18.49)	<.0001	146 (7.71)	20 (8.62)	93 (12.13)	0.0014
2–3 h	125 (8.09)	13 (5.99)	83 (13.65)	<.0001	58 (6.24)	10 (7.04)	43 (11.75)	0.0036
≥4 h	22 (3.88)	2 (2.13)	15 (8.47)	0.0189	21 (6.12)	0 (0)	10 (8.93)	0.0680
Doing or helping with house work								
Never	160 (22.22)	23 (20.18)	116 (29.90)	0.0096	33 (9.02)	6 (10.53)	31 (15.82)	0.0517
Occasionally	252 (11.09)	41 (12.89)	173 (18.21)	<.0001	111 (7.61)	22 (10.38)	73 (12.56)	0.0017
Regularly	352 (10.30)	36 (8.13)	220 (16.98)	<.0001	139 (6.34)	22 (7.24)	90 (11.28)	<.0001

### Incidence of frailty

A total of 7,314 older adults were followed up at 12 months, of which 6,293 were eligible subjects (Table 1). At one year follow-up, 527 new cases of frailty were detected, and the overall incidence of frailty increased from non-diabetes to pre-diabetes and diabetes group (7.04, 8.73, 12.32%, p < 0.001). Such increasing trends were also found in other demographic and clinical subpopulations. For instance, the incidence in females were increased by glucose status (7.87, 8.84, 14.10%) compared to those in the males (5.82, 8.57, 9.65%) in non-diabetic, pre-diabetic and diabetic subjects respectively. For age group between 55 to 64, the incidence of frailty was the lowest compared with other age groups, but the incidence in pre-diabetic and diabetic subjects were both significantly higher than subjects with normal glucose, 2.76% vs. 4.44% vs. 4.62%, respectively. For subgroups aged 65–74 and 75–84 years, the incidence also increased from 6.36% to 9.24 and 11.22% and from 12.17% to 13.14 and 20.33%, respectively. For the oldest age group, there was not enough data in pre-diabetic subjects to show the increasing trend, but the incidence rate in diabetic subjects was much higher than non-diabetes (42.11% vs. 25.32%).

### Crude and adjusted risks of frailty comparing pre-diabetic and diabetic subjects with those with normal blood glucose at baseline and follow-up survey

Using both unadjusted and adjusted logistic regression, we found having diabetes increased the risk of prevalence frailty at baseline (Additional file 2: Table S2 Appendix 2), the crude and adjusted OR were 1.77 (95%CI = 1.56–2.00, *P* < .0001), and 1.36 (95%CI = 1.18–1.56, *P* < .0001), controlling for age, sex, residency and co-morbidity. However, the prevalence of frailty in pre-diabetics was not statistically higher than those in non-diabetics (unadj. OR = 0.95, adj. OR = 0.96). For longitudinal risk of frailty at one-year follow-up visit, subjects with diabetes were statistically higher than those with normal blood glucose using both unadjusted RR (1.75, 95%CI = 1.47–2.08, *p* < 0.001) and adjusted RR by age, sex, residency and comorbidity (adj. RR = 1.56, 95%CI = 1.32–1.85, *p* < .0001). Pre-diabetics also showed an increased risk of frailty, the unadjusted RR and adjusted RR were 1.24 (95%CI = 0.93–1.65, *p* = 0.1439) and 1.28 (95%CI = 0.96–1.70, *p* = 0.0886) respectively, however didn't reach the statistical significant level.

### Associated factors for risk of prevalence of frailty in elevated blood glucose (pre-diabetic and diabetic) population and normal blood glucose (non-diabetic) population

In Table 2, we stratified the baseline participants by blood glucose level status, ie. elevated blood glucose (pre-diabetes and diabetes) and normal blood glucose (non-diabetic) and developed multiple logistic model of frailty for each stratum. Both strata shared four common risk factors and three protective factors for frailty, including urban living (adj. ORs = 2.29 and 3.07 in elevated and normal glucose group, respectively), less than 6-h sleep per day (adj. ORs = 1.56 and 1.48), with comorbidity (adj. ORs = 6.33 and 11.82 for having 1–2 diseases; 43.56 and 60.46 for having 3 or more diseases), polypharmacy (adj. ORs = 3.44 and 2.50), and being married (adj. ORs = 0.60 and 0.66, more hours of outdoor activity (adj. ORs = 0.24 to 0.20 and 0.23 to 0.19) and helping with house work regularly (adj. ORs = 0.56 and 0.43). Three more risk factors were found in pre-diabetic and diabetic subgroup, such as low education (adj. OR = 1.60), irregular life style (adj. OR = 4.74), not often drinking milk (adj. OR = 1.61). Being female and older age statistically increased the prevalence of frailty only in non-diabetes subpopulation.

**Table 2:** Backward elimination variable selection method to investigate if the elevated blood glucose (pre-diabetes and diabetes) frail population shared common risk factors as other frail population at baseline

	Prevalence of frailty in pre-diabetic and diabetic subjects (*n* = 3509)			Prevalence of frailty in subjects with normal blood glucose (*n* = 6530)		
	Adj. OR	(95%CI)	P value	Adj. OR	(95%CI)	*P* value
Male vs female	/	/	/	0.66	(0.50,0.87)	0.0031
Currently married vs not	0.60	(0.43,0.85)	0.0041	0.66	(0.50,0.88)	0.0052
Urban vs rural	2.29	(1.30,4.05)	0.0022	3.07	(1.85,5.09)	<.0001
Age group						
55–64	/	/	/	Ref	Ref	Ref
65–74	/	/	/	1.60	(1.09,2.37)	0.0649
75–84	/	/	/	2.33	(1.56,3.49)	0.1907
≥85	/	/	/	4.25	(2.04,8.83)	0.0026
Low education vs higher	1.60	(1.18,2.15)	0.0022	/	/	/
Not regular lifestyle vs regular	4.74	(2.02,11.14)	0.0004	/	/	/
Not Regularly drinking milk vs regular	1.61	(1.19,2.18)	0.0020	/	/	/
Less than 6 h/day sleep vs more sleep	1.56	(1.11,2.20)	0.0100	1.48	(1.12,1.96)	0.0053
Number of comorbidity^a^						
0	Ref	Ref	Ref	Ref	Ref	Ref
1–2	6.33	(2.70,14.80)	0.8612	11.82	(5.49, 25.42)	0.0489
≥3	43.56	(18.40,103.16)	<.0001	60.46	(27.72, 131.88)	<.0001
Polypharmacy ≥4 types/day	3.44	(2.57,4.62)	<.0001	2.50	(1.93,3.22)	<.0001
Hours of outdoor activity daily						
None	Ref	Ref	Ref	Ref	Ref	Ref
0.5–1 h	0.31	(0.17,0.54)	0.3381	0.26	(0.17, 0.41)	0.0660
2–3 h	0.24	(0.12,0.45)	0.0247	0.229	(0.14, 0.38)	0.0129
≥4 h	0.20	(0.08,0.50)	0.0559	0.190	(0.09,0.40)	0.0291
Doing or helping with house work						
Never	Ref	Ref	Ref	Ref	Ref	Ref
Occasionally	0.80	(0.52,1.24)	0.6714	0.66	(0.44, 0.97)	0.9925
Regularly	0.56	(0.37,0.85)	0.0029	0.43	(0.29, 0.63)	<.0001

^a^Number of comorbidity include any of the 11 chronic conditions in the FI

### Associated factors for risk of incidence of frailty in elevated blood glucose (pre-diabetic and diabetic) population and normal blood glucose (non-diabetic) population

In Table **3**, we investigated the risk factors of incidence of frailty longitudinally by blood glucose status subpopulation using separate multiple logistic models. The common risk factors for frailty incidence in both populations were age (adj. RR = 1.70, 2.62, 4.88 and 2.01, 3.49, 7.72 in elevated and normal glucose groups, respectively), and comorbidity (adj. RR = 1.19 and 2.13 for 1–2 diseases, 3.02 and 3.43 for 3 or more diseases, respectively); and the common protective factor was receiving regular medical consultation (adj. RR = 0.79 and 0.72 in elevated and normal glucose groups, respectively). Two risk factors including urban-living (adj. RR = 2.28), higher waist circumference (adj. RR = 1.37) and two protective factors including being male (adj. RR = 0.67),doing house work regularly (adj. RR = 0.67) were only among pre-diabetes and diabetes subjects. For the non-diabetic participants polypharmacy was a risk factor of frailty (adj. RR = 1.44) and awareness of their blood glucose status was protective against frailty (adj. RR = 0.72).

**Table 3:** Backward elimination variable selection method to investigate if the elevated blood glucose (pre-diabetes and diabetes) frail population shared common risk factors as other frail population at one-year follow-up

	Incidence of frailty in pre-diabetes and diabetes subjects (*n* = 2176)		Incidence of frailty in subjects with normal blood glucose (*n* = 4117)	
	Adj. RR (95%CI)	*P* value*	Adj. RR (95%CI)	*P* value*
Male vs female	0.67 (0.52,0.86)	0.0017	/	/
Urban vs rural	2.28 (1.32, 3.91)	0.0030	/	/
Age group				
55–64	Ref	Ref	Ref	Ref
65–74	1.70 (1.12, 2.58)	0.0125	2.01 (1.37,2.93)	0.0003
75–84	2.62 (1.71, 4.03)	<.0001	3.49 (2.38,5.12)	<.0001
≥85	4.88 (2.53, 9.42)	<.0001	7.72 (4.61,12.94)	<.0001
Regularly receiving medical consultation after baseline vs not receiving	0.79 (0.62,1.00)	0.0463	0.72 (0.58, 0.90)	0.0041
Regular anti diabetic treatment vs irregular	1.16 (0.91, 1.48)	0.2240	/	/
Number of comorbidity^a^				
0	Ref	Ref	Ref	Ref
1–2	1.19 (0.83,1.70)	0.3386	2.13 (1.53, 2.98)	<.0001
≥3	3.02 (2.07,4.39)	<.0001	3.43 (2.30, 5.12)	<.0001
Polypharmacy ≥4 types/day	/	/	1.44 (1.11, 1.86)	0.0062
Doing or helping with house work				
Never	Ref	Ref	/	/
Occasionally	0.87 (0.61,1.24)	0.4344	/	/
Regularly	0.67 (0.47,0.95)	0.0247	/	/
Waist circumference high vs normal	1.37 (0.99,1.89)	0.0549	/	/
Self-awareness of blood glucose level	/	/	0.72 (0.57, 0.90)	0.0036

*Number of comorbidity include any of the 11 chronic conditions in the FI

### Discussion

Our results for the overall prevalence and incidence of frailty in the population without diabetes and with diabetes are consistent with previous studies [6–8, 12, 19–23]. Both rates were found to be the most lowest in older population without diabetes, slightly higher in pre-diabetic and highest in diabetic population. Such increasing trends of frailty by blood glucose level were similarly found in age and other demographic subgroups, showing diabetes is an independent predictor of the prevalence and incidence of frailty. Whereas, pre-diabetes or impaired fasting glucose although not very distinct could be playing an intermediary role along with other risk factors in the acceleration of frailty. Therefore, attention should be given to the elderly population with impaired fasting glucose levels as well. Older adults with more co-morbidity and taking more medications were found to have a very high prevalence and incidence of frailty in all three groups (ie non-diabetics, pre-diabetics and diabetics), indicating that frailty is severely affected by multi co-morbidity and polypharmacy, which is in consistent with previous studies [6–8, 19–23], and if were additionally to be affected with pre-diabetes or diabetes, the condition could worsen [24, 25]. More importantly, elderly population with pre-diabetes are known to have low grade systemic inflammation and oxidative stress [25], leading to metabolic dysfunction which may affect the components of physical frailty, therefore should not be ignored. Therefore, physicians should advise older adults to follow proper blood sugar control techniques at an early phase of abnormal blood glucose status.

Elevated blood glucose populations shared some common risk factors as non-diabetic populations in both cross-sectional and longitudinal analysis. At baseline, the following subgroups demonstrated higher prevalence of frailty despite of blood glucose status: not being married, urban residency, daily sleep less than 6 h, co-morbidity, polypharmacy, less outdoor activity time and rarely helping with house work. To screen more people with frailty in community, elderly subjects with above characteristics should be targeted. However, lower education and irregular life style and less intake of milk were only correlated with the prevalence of frailty in pre-diabetic and diabetic subjects. In contrast, advanced age and being female were positively associated with frailty only among subjects with normal blood glucose. In light of this, health care providers should consider screening for frailty of at-risk older people and followed by targeted interventions for deficit or risk issues. The different sets of risk factors for normal and elevated blood glucose subgroups have been identified using longitudinal data. Among pre-diabetic and diabetic older subjects, the unmodifiable risk factors were female sex, urban living, and modifiable risk factors were higher waist-circumference, less house work and in need of taking regular anti-diabetic medication. Among older adults with normal blood glucose, only poly-pharmacy and unawareness of blood glucose were found to be independent risk factor of developing frailty. Therefore, the interventions in community health centers among non-diabetic subjects should target on consultation on polypharmacy prescription and improving individual's awareness of blood glucose level. Considering the serious and worse adverse events among frail older people with diabetes, screening and intervention of frailty should be proactively implemented as soon as pre-diabetes is detected.

Consistent with previous studies, doing regular household work and performing more outdoor activity had positive effect for frail older adults [26–28]. Therefore, routinely proper physical activities, participation in more house work and losing waist for those with higher waist circumference should be an urgent need to prevent frailty in elderly subjects [29–31]. Our study has also demonstrated some more protective factors of frailty, such as drinking milk, maintaining regular daily life-style and awareness blood glucose level and getting routinely geriatric health consultation. Geriatricians and physicians should take in account of these factors while advising the older patients.

Our study is the first to show the relationship of elevated blood glucose status with frailty in Chinese older adults. However, some limitations of this study are worth mentioning: we used fasting plasma glucose, personal history of diagnosis of diabetes and use of anti-diabetic medication as the diagnosis criteria for pre-diabetes and diabetes, oral glucose tolerance testing was not taken (as it is in-practicable for such a large cohort) and glycosylated hemoglobin (HbA1c) levels were not measured which might have misclassified some of the participants. Future studies in the subject could focus in the limitation of this study. And more studies are warranted on dose–response relationship and their etiology rationale between blood glucose level and frailty, as well as cost-effectiveness analyses on managing frailty among pre-diabetes and diabetes in various community settings.

## Conclusion

This study confirms that diabetes is an independent serious chronic condition in older adults to increase the risk of frailty. Highest prevalence and incidence of frailty is observed in elderly population with diabetes. Frailty in elevated blood glucose older adults shares common risk factors, as other non-diabetic frail older population. Being female, urban living, high waist circumference, less house work and need regular anti-diabetic medications are independent risk factors only in pre-diabetic and diabetic older adults. Geriatricians should advise the older patients both with elevated and normal blood glucose levels by different strategies individually. Additionally, results of this study could facilitate future studies in identification of the at-risk community dwelling older subjects and implementation of suitable interventions for frailty.

## Additional files

Additional file 1: Table S1. Characteristics of study population. (DOCX 33 kb)
Additional file 2: Table S2. Odds ratios and relative risks of frailty comparing pre-diabetic and diabetic subjects with those with normal blood glucose at baseline and follow-up visits. (DOCX 29 kb)
