# Supplementary material for: The prevalence and incidence of frailty in Pre-diabetic and diabetic community-dwelling older population: results from Beijing longitudinal study of aging II (BLSA-II)
Source: BMC Geriatr. 2017 Feb 8;17:47. doi: 10.1186/s12877-017-0439-y (PMC5299771; doi:10.1186/s12877-017-0439-y)
Supplement: Additional file 1: Table S1. — Characteristics of study population. (DOCX 33 kb) [file 12877_2017_439_MOESM1_ESM.docx]

***Supplementary Table 1:***

Characteristics of study population

|  | **Baseline (2010)** | | | **One-year Follow-up (2011)** | | |
| --- | --- | --- | --- | --- | --- | --- |
|  | **Male**  **(n=3886)**  **N(%)** | **Female**  **(n=6153)**  **N(%)** | **Total**  **( n=10039)**  **N(%)** | **Male**  **(n=2576)N(%)** | **Female**  **(n=3717)**  **N(%)** | **Total**  **(n=6293)**  **N(%)** |
| **Age** | 70.94 ± 7.90 | 70.24 ± 7.76 | 70.51 ± 7.82 | 70.60 ±7.54 | 69.72±7.15 | 70.08±7.33 |
| 55-64 (n=2741) | 1035 (26.63) | 1706(27.73) | 2741(27.30) | 709(27.52) | 1092(29.38) | 1801(28.62) |
| 65-74 (n=4266) | 1549 (39.86) | 2717(44.16) | 4266(42.49) | 1073(41.65) | 1741(46.84) | 2814(44.72) |
| 75-84 (n=2785) | 1183 (30.44) | 1602(26.04) | 2785(27.74) | 735(28.53) | 830(22.33) | 1565(24.87) |
| ≥85 (n=247) | 119(3.06) | 128(2.08) | 247(2.46) | 59(2.29) | 54(1.45) | 113(1.80) |
| **Education** |  |  |  |  |  |  |
| Less than middle school | 1020 (26.28) | 2847(46.29) | 3867(38.55) | 651(25.30) | 1649(44.38) | 2300(36.57) |
| Middle school or higher | 2861 (73.72) | 3303(53.71) | 6164(61.45) | 1922 (74.70) | 2067(55.62) | 3989(63.43) |
| **History of Smoking** |  |  |  |  |  |  |
| Yes | 1054 (27.12) | 254(4.13) | 1308(13.03) | 702(27.25) | 148(3.98) | 850(13.51) |
| No | 2832 (72.88) | 5899 (95.87) | 8731(86.97) | 1874 (72.75) | 3569 (96.02) | 5443(86.49) |
| **History of Drinking** |  |  |  |  |  |  |
| Yes | 1023 (26.33) | 114 (1.85) | 1137 (11.33) | 673 (26.13) | 74 (1.99) | 747(11.87) |
| No | 2863 (73.67) | 6039 (98.15) | 8902 (88.67) | 1903 (73.87) | 3643(98.01) | 5546(88.13) |
| **Taking≥4 medication** |  |  |  |  |  |  |
| Yes | 826(21.46) | 1392 (22.89) | 2218 (22.34) | 469(18.31) | 676(18.39) | 1145(18.36) |
| No | 3023 (78.54) | 4689 (77.11) | 7712 (77.66) | 2093 (81.69) | 2999(81.61) | 5092(81.64) |
| **Number of Chronic diseases** |  |  |  |  |  |  |
| 0 | 1130 (29.08) | 1453 (23.61) | 2583 (25.73) | 779(30.24) | 954(25.67) | 1733(27.54) |
| 1 – 2 | 2040 (52.50) | 3331 (54.14) | 5371 (53.50) | 1415(54.93) | 2153(57.92) | 3568(56.70) |
| ≥3 | 716(18.43) | 1369 (22.25) | 2085(20.77) | 382(14.83) | 610(16.41) | 992(15.76) |
| **Residence** |  |  |  |  |  |  |
| Rural | 3033 (78.05) | 4842 (78.69) | 2164 (21.56) | 534(20.73) | 741(19.94) | 1275(20.26) |
| Urban | 853(21.95) | 1311 (21.31) | 7875 (78.44) | 2042 (79.27) | 2976(80.06) | 5018(79.74) |
| **Hours of outdoor activity daily** |  |  |  |  |  |  |
| None | 178(4.59) | 293 (4.78) | 471 (4.71) | 92(3.58) | 113(3.05) | 205(3.27) |
| ＜0.5hr | 698(18.01) | 1106 (18.04) | 1804 (18.03) | 455(17.72) | 681(18.38) | 1136(18.11) |
| 0.5-1hour | 1745(45.03) | 275 (44.92) | 4499 (44.96) | 1210 (47.14) | 1744(47.07) | 2954(47.10) |
| 2-3hours | 866(22.35) | 1521(24.81) | 2387(23.86) | 563(21.93) | 891(24.05) | 1454(23.18) |
| ≥4 hours | 388(10.01) | 457(7.45) | 845(8.44) | 247(9.62) | 276(7.45) | 523(8.34) |
| **Doing or helping with house work** |  |  |  |  |  |  |
| Never | 673(17.32) | 567(9.22) | 1240(12.35) | 382(14.83) | 260(6.99) | 642(10.20) |
| Occasionally | 1797(46.24) | 1783(28.98) | 3580(35.66) | 1194 (46.35) | 1117(30.05) | 2311(36.72) |
| Regularly | 1416(36.44) | 3803(61.81) | 5219(51.99) | 1000 (38.82) | 2340(62.95) | 3340(53.07) |
