# Supplementary material for: The prevalence and incidence of frailty in Pre-diabetic and diabetic community-dwelling older population: results from Beijing longitudinal study of aging II (BLSA-II)
Source: BMC Geriatr. 2017 Feb 8;17:47. doi: 10.1186/s12877-017-0439-y (PMC5299771; doi:10.1186/s12877-017-0439-y)
Supplement: Additional file 2: Table S2. — Odds ratios and relative risks of frailty comparing pre-diabetic and diabetic subjects with those with normal blood glucose at baseline and follow-up visits. (DOCX 29 kb) [file 12877_2017_439_MOESM2_ESM.docx]

***Supplementary Table 2***

Odds ratios and relative risks of frailty comparing pre-diabetic and diabetic subjects with those with normal blood glucose at baseline and follow-up visits

|  | Adj.OR  (95%CI)* | P value | Adj.RR  (95%CI)* | P value |
| --- | --- | --- | --- | --- |
| Pre-diabetes vs.non-diabetes | 0.96 (0.75, 1.23) | 0.127 | 1.28 (0.96,1.70) | 0.0886 |
| Diabetes vs. non-diabetes | 1.36 (1.18, 1.56) | 0.0001 | 1.56 (1.32,1.85) | <.0001 |
| Male | 0.71 (0.62, 0.82) | <.0001 | 0.71 (0.60,0.84) | <.0001 |
| Urban | 2.22 (1.72, 2.86) | <.0001 | 1.37 (1.01,1.84) | 0.0403 |
| Age group |  |  |  |  |
| 55-64 | Ref | Ref | Ref | Ref |
| 65-74 | 1.70 (1.37, 2.11) | <.0001 | 1.90 (1.43,2.53) | <.0001 |
| 75-84 | 3.11 (2.50,3.87) | <.0001 | 3.25 (2.42,4.37) | <.0001 |
| ≥85 | 5.90 (4.07,8.55) | <.0001 | 7.08 (4.75, 10.55) | <.0001 |
| Comorbidity** |  |  |  |  |
| 0 | Ref | Ref | Ref | Ref |
| 1-2 | 10.11 (6.89,14.84) | 0.008 | 1.72 (1.35, 2.19) | <.0001 |
| ≥. | 57.70 (39.15,85.03) | <.0001 | 3.77 (2.89,4.90) | <.0001 |

*after adjusting for age, sex, residency and comorbidity, **Number of comorbidity include any of the 11 chronic conditions in the FI
